# Novel Magnetic Sensing Approach with Improved Linearity

**DOI:** 10.3390/s130607618

**Published:** 2013-06-13

**Authors:** Marco Fontana, Fabio Salsedo, Massimo Bergamasco

**Affiliations:** PERCRO Laboratory, TeCIP Institute, Scuola Superiore Sant'Anna, Piazza Martiri della Libertà 33, Pisa 56127, Italy; E-Mails: f.salsedo@sssup.it (F.S.); m.bergamasco@sssup.it (M.B.)

**Keywords:** angle sensor, Hall effect, magnetic charge, low cost, rotation sensor, diametrical magnetization, permanent magnet

## Abstract

This paper introduces a novel contactless sensing principle conceived for measuring the rotation angle of a shaft. The sensor is based on a smart combination of low-cost components that can be effectively integrated in a mechanical assembly of a rotary joint. The working principle is based on the relative rotation of a small diametrically magnetized cylindrical or annular magnet and at least one Hall effect sensor. One of the main strengths of the new sensing principle is to be adaptable to any assigned dimensions and encumbrances without typical design limitations given by the use of standard components. A numerical model is developed for predicting the sensor output characteristic on the base of the concept of magnetic charge. Such a model is validated against results from laboratory experiments. The parameters that define the geometry and layout of the sensor are optimized in order to maximize linearity over an assigned angular range of measurement. Two examples of mechatronic systems that employ the new sensing principle are presented in order to show the possibility of obtaining with the new principle a compact/integrated sensor-design.

## 1. Introduction

Angle sensors are devices designed to transduce the relative rotation of two bodies into electrical signals. Many different basic principles have been developed in the past and many commercial products are available on the market. A commercial sensor is generally available in the form of an assembly that already possesses its own rotation shaft, bearings (or bushes), enclosure and wiring. Optical/magnetic encoders or potentiometers are among the most common of this kind of sensors. However, in a number of application fields of mechatronics, angle sensors are required to be deeply integrated and incorporated into mechanical structures. In such cases the use of commercial sensors is not always viable because of:

- integration problems: the sensor has its own structure (shaft, enclosure, bushes, *etc.*) and the coupling with an existing rotation axis can be trivial and might require additional components and spaces;
- compactness: the integration of the sensor assembly that has its own standard dimensions and encumbrances, usually causes the mechanical encumbrances of the assembly to increase;
- cost: if the sensor has it's own rotation axis additional mechanical components are required for a proper connection with the rotation axis. The manufacturing of this components increase the cost of the whole assembly;
- reliability: the use of additional components may generally reduce the reliability of the system.

Alternatively, the angle sensor can be purposely “designed around” the existing mechanical structure (shafts or leverages) to fit dimension and cost requirements. A very common solution, which is adopted in these cases, is to employ non-contact magnetic sensors in combination with a moving/rotating magnetic field generated by a permanent magnet or a coil. A linear Hall Sensor (HS) is a very good candidate among the magnetic sensors because it presents a good balance between: accuracy, robustness/reliability, resistance to dirty/harsh environments, low cost, manufacturability, reduced required encumbrances and easiness of wiring. A first example of angle sensor that employs HS was developed by Strandt [1] and, more recently, HS find applications in many different fields such as: the automotive sector (fuel level float-arm rotation, window displacement, force sensing of the steering bar [2]), aeronautics (displacement based torque sensors, rpm sensors [3]), electric actuators [4,5] and robotics [6,7]. A basic explanation of the working principle of linear HS is provided by Lenz in [8]. In the last few decades, researchers and engineers have conceived a number of different solutions for angle measurement sensors that combine magnets, HS and ferromagnetic components. Different layouts have been analyzed with the aim of improving sensing performances according to specific application needs. In several designs the output of the shaft angle sensor has sinusoidal-like output and multiple HS are employed to provide 360° of measuring range and absolute angle sensing [9,10]. In other designs, miniaturization is highly sought-after and custom CMOS circuits that integrate miniaturized HS are purposely developed [11–13]. Other designs are conceived for optimizing resolution by using large number of HS [14]

In this work, we focus particularly on sensing approaches that provide linear sensing responses. Linearity, although over a limited angular range, can be very desirable in different applications since it allows one to integrate the sensor without the need of characterization and/or collection of data for the preparation of look-up tables. Linear response can be obtained in small angular range by the employment of traditional solutions that provide a sinusoidal-like output [15]. Differently, purposely-developed solutions can be employed to provide an extended linear range. A special oval-shaped magnet has been developed by Lemarquand [16] in order to reduce the linearity error over a large range of measurement and Schott in [17] presents a method for obtaining liner output by employing smartly-arranged magneto-resistive transducers. In other studies, linearity is obtained through the implementation of a variable reluctance sensor [18]. Of course, extended measurement ranges can be alternatively obtained by interpolation of sensor output stored in look-up tables. However, even in this case, the linearity of the sensor can be advantageous in order to have a uniform resolution after the digitalization of the signal.

In this paper, we propose a simple linear sensing solution that employs common commercial components such as a diametrically magnetized flat cylindrical or annular permanent magnet and a HS. The layout of the sensing principle is conceived to provide a linear response-curve within a limited range of measurement. The sensor is based on simple components that can be custom built according to specific requirements. One of the main strengths of the obtained sensing principle is the possibility of being adapted to assigned spaces and encumbrances. This makes it extremely suitable for robotic and mechatronic prototyping when limited angular ranges and simple/compact/integrated design are highly desired and where the requirement of minimizing electrical cabling is crucial.

In order to evaluate the performance of sensing, a numerical model for the estimation of the magnetic field produced by the magnet is developed and presented in the second section of this paper. Such a model is based on the concept of the magnetic charge [19] that was employed by Ravaud *et al.* [20] to find an analytical solution for the magnetic field created by permanent-magnet rings that are axially and radially magnetized. In this work, we introduce a partial analytical solution for diametrically magnetized cylindrical magnets with finite axial length, which has not been analyzed in literature.

In the third section of the paper, we show a design procedure that provides an optimal combination of geometrical variables of the sensor starting from any given dimension of the magnet. The procedure aims at maximizing the linearity of the output of the sensor over a specified measuring range. Several layouts characterized by different measuring ranges and number of HS are analyzed.

In the fourth section, we report measurement and experiments that have been conducted to validate the developed models. In the last section of the paper we show two examples of devices that employ the new sensing principle. The first one is a mechanical tracker described in [21] used in combination with a haptic hand-exoskeleton [22], the second is a robotic rehabilitation device that is under development at the time we are writing [23].

## 2. Model of a Diametrically Magnetized Cylindrical/Annular Permanent-Magnet

**Figure 1.**
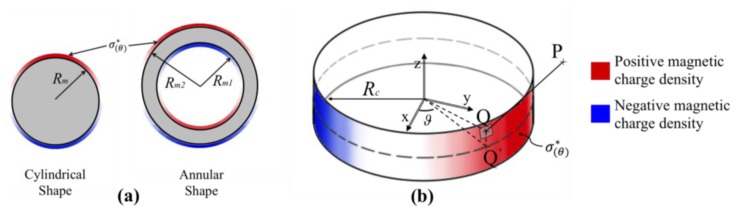
(**a**) Scheme of the distribution of the equivalent magnetic charge on the cylindrical surfaces of the cylindrical and annular magnets; (**b**) scheme of the geometry of the model employed for the solution.

In order to evaluate the output of the new proposed sensing principle we have developed a method for the estimation of the sensor output based on analytical/numerical analysis. Magnetic field generated by permanent magnets can be computed with the use of the Couloumbian model (also known as magnetic charge method). Ravaud in [20] employs such method for analyzing the problem of the magnetic field generated by annular permanent magnets that are axially or radially magnetized. In such cases, the magnetization vector *M⃗* is normal to the surface of the permanent magnet thus the magnetic charge is uniformly distributed on the boundary surfaces. For a diametrical magnetization such hypothesis is not valid since *M⃗* forms inconstant angle with *n̂*, normal unit vector to the boundary surface. In this case, with reference to Figure 1, the magnetic charge $\sigma_{\left(\theta \right)}^{*}$ can be written in function of the angle *θ* as:

(1) $$\sigma_{\left(\theta \right)}^{*}=\vec{M}\cdot \overset{⌢}{n}=M\sin(\theta )$$

where *M⃗* is assumed to be oriented along the *y*-axis. It is important to underline that Equation (1) is valid under the following assumptions [24]:

- permanent magnet has uniform, constant and isotropic magnetic permeability and its value is close to air permeability (practically valid for rare earth magnets);
- there are no distortion on the magnetic field caused by surrounding ferromagnetic components;
- the magnetization field in the permanent magnetic material is uniform and constant.

Under these assumptions, the magnetic field *H⃗* can be computed, in the case of a cylindrical magnet, at any observation point *P* using cylindrical coordinates (*ρ, ϕ, z*) with azimuth coincident with the *x*-axis:

(2) $$\begin{array}{l} {\vec{H}}_{\left(\rho ,\phi ,z\right)}=\frac{1}{4\pi \mu_{o}}\int_{\theta =-\pi }^{\theta =\pi }\int_{z_{Q}=-h/2}^{z_{Q}=+h/2}\frac{\vec{QP}}{{\left|\vec{QP}\right|}^{3}}\sigma_{\left(\theta \right)}^{*}\;dz_{Q}\;R_{c}\;d\theta \\ \;=\frac{M}{4\pi \mu_{o}}\int_{\theta =-\pi }^{\theta =\pi }\int_{z_{Q}=-h/2}^{z_{Q}=+h/2}\frac{\vec{QP}}{{\left|\vec{QP}\right|}^{3}}\sin(\theta )dz_{Q}R_{c}d\theta , \end{array}$$

where *Q*_(*R_c_*, *θ*, *z_Q_*)_ is a point on the cylindrical surface of the magnet, *R_c_* is the radius of the permanent magnet and *h* is the axial length of the magnet.

Equation (2) can be numerically solved and a partial analytical solution can be found for certain positions of *P*. In particular, if the point *P* belongs to the symmetry plane *z* = 0, the Equation (2) can be partially analytically solved. Given that, the charge density is not depending on the and the domain of the rightmost integral of Equation (2) do not depends on the variable *θ*, the rightmost integral can be independently solved referring to simple known integral forms. In particular, Equation (2) simplifies to:

(3) $$\begin{array}{l} {\vec{H}}_{\left(\rho ,\phi ,z=0\right)}=\frac{M}{4\pi \mu_{0}}\int_{\theta =-\pi }^{\theta =\pi }\sin(\theta )\left|\int_{Z_{Q}=-h/2}^{Z_{Q}=+h/2}\frac{\vec{QP}}{{\left|\vec{QP}\right|}^{3}}dz_{Q}\right|R_{c}\;d\theta =\\ \;=\frac{M\;h\;R_{c}}{4\pi \mu_{0}}\int_{\theta =-\pi }^{\theta =\pi }\frac{\sin\left(\theta \right)\vec{Q\prime P}}{{\left|\vec{Q\prime P}\right|}^{2}\sqrt{{\left(\frac{h}{2}\right)}^{2}+{\left|\vec{Q\prime P}\right|}^{2}}}d\theta \end{array}$$

that is obtained integrating over *z* in the interval [−*h*/2;*h*/2] and indicating with *Q*′ the projection of *Q* on the *x-y* plane. Without providing the complete analytical solution, it is anyway possible to write the radial and tangential components of the magnetic field through a set of simplifications. By substituting θ* = *θ* − *α* in Equation (3) and by considering that the integrand is composed by a combination of periodic odd/even functions integrated over the interval [−*π*; *π*], we can write the resulting components of the magnetic field as follow:

(4) $$\begin{array}{l} {\vec{H}}_{\rho }=\left[\frac{MhR_{c}}{4\pi \mu_{o}}\int_{\theta *=-\pi }^{\theta *=\pi }\frac{\rho \cos\left(\theta *\right)-R_{c}\cos^{2}\left(\theta *\right)}{{\left|\vec{Q\prime P_{\left(\theta *\right)}}\right|}^{2}\sqrt{{\left(\frac{h}{2}\right)}^{2}+{\left|\vec{Q\prime P_{\left(\theta *\right)}}\right|}^{2}}}d\theta *\right]\sin(\phi ),\\ \begin{array}{l} {\vec{H}}_{\phi }=\left[\frac{MhR_{c}}{4\pi \mu_{o}}\int_{\theta *=-\pi }^{\theta *=\pi }\frac{-R_{c}\sin^{2}\left(\theta *\right)}{{\left|\vec{Q\prime P_{\left(\theta *\right)}}\right|}^{2}\sqrt{{\left(\frac{h}{2}\right)}^{2}+{\left|\vec{Q\prime P_{\left(\theta *\right)}}\right|}^{2}}}d\theta *\right]\\ \;{\vec{H}}_{z}=0. \end{array}\cos(\phi ), \end{array}$$

which show a behavior that resembles the magnetic field of a dipole having radial component that depends on the sine of the azimuth angle and tangential component depending on the cosine.

The full analytical solution might be found with the approaches presented in [25] considering the additional difficulty given by the limited depth of the magnet. However, although it could be an interesting topic to treat, this dissertation is out of the scope of this paper.

It is important to remark that Equation (4) can be applied also for the computation of the magnetic field for an annular shaped permanent magnet. The field in this case is computed by the application of the linear superposition principle, as the sum of the fields generated by two cylindrical magnets: the first with a diameter equal to the external diameter of the annular shape and with a magnetization equal to the real magnet; the second with a diameter equal to the internal diameter of the annular magnet but with a magnetization that is opposed in direction to the original one [see Figure 1(a)].

## 3. Layout and Design Optimization

**Figure 2.**
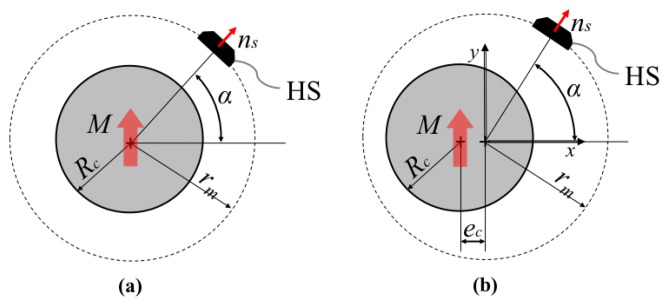
Layout of the sensing principle (**a**) traditional sensing with sinusoidal-like output (**b**) modified layout with eccentricity.

The layout of the proposed sensor is based on a minor geometrical modification of a well-known scheme that employs a cylindrical/annular magnet that rotates around its own axis and one HS whose sensing direction is: (1) oriented towards the magnet axis and (2) lays on the symmetry plane that is normal to the magnet axis (see Figure 2(a)). Such a sensor configuration presents many positive features such as extreme low cost, absolute sensing and easy adaptation/customization to a given geometry. However, the output from a single HS is theoretically sinusoidal and consequently it can be hardly employed for linear angle measurement. If linearity errors are tolerable, it can be employed as linear sensor in a very reduced range of motion but in any case the range cannot exceed ±90° without using multiple HS.

The sensor that is described in the following sections is conceived to retain the minimalism and simplicity of the layout of this sensing principle, but with improved linear range of measurement given by a smart arrangement of components.

### 3.1. Working Principle

The proposed sensing principle is based on the introduction of a misalignment between the axis of the circular/annular magnet and the axis of relative rotation between the magnet and the HS. If we assume the magnet at rest, the HS moves on a circular trajectory of radius *r_m_*(radius of measurement) centered in the point [*e_c_*,0,0]as represented in Figure 2(b).

**Figure 3.**
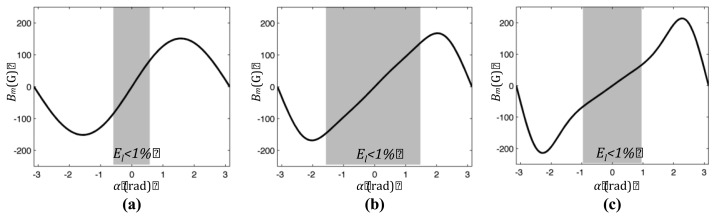
Estimated measured radial component of component *B⃗* *versus* angle. Plots refer to cylindrical magnet with *M⃗* = [0,1*T*,0], radius R_c_ = 6 mm, thickness *h* = 6 mm, measurement radius is *ρ* = 10mm and eccentricity values (**a**) *e_c_* = 0; (**b**) *e_c_* = 1 mm; (**c**) *e_c_* = 2 mm

The magnetic field can be calculated at any point P_s_ of the *x-y* plane (symmetry plane normal to the magnet axis) by integration of Equation (4). The component of the magnetic field along the sensitive direction of the HS can be computed in each point of the circular trajectory *P_s_*_(*r_m_*, *α*)_ (the dependency from *z* component is omitted since we refer to points that belongs to the *x-y* plane) as:

(5) $$B_{m(r_{m},\alpha )}={\vec{B}}_{\left(r_{m},\alpha \right)}.{\overset{⌢}{n}}_{s(r_{m},\alpha )}$$

where *B_m_*_(*r_m_*, *α*)_ is the estimation of the measured component of the induction vector, *B⃗*_(*r_m_*, *α*)_ is the magnetic induction that is obtained dividing the *H⃗* field (Equation (4)) computed at the point *P_s_*_(*r_m_*, *α*)_ by the vacuum permeability, and *n̂_s_*_(*r_m_*,*α*)_ is the unit vector representing the sensitive direction of the HS. The effect of the introduction of an increasing value of eccentricity is shown in Figure 3. As it can be qualitative observed, the response curve has a slightly increased linear field for particular values of the eccentricity.

The estimated linearity error (*E_l_*) in a specific measuring range (*α_m_*) can be computed as the maximum deviation between the estimated curve of *B_m_* and the linear least square fitting line (*B_l_*) computed over the specified range of data [−*α_m_*; *α_m_*], divided by the maximum value of *B_m_*:

(6) $$E_{l}=\underset{\alpha =[-\alpha_{m};\alpha_{m}]}{\max}\;\left(\frac{\left(B_{m(\alpha )}-B_{l(\alpha )}\right)}{\underset{\alpha =[-\alpha_{m};\alpha_{m}]}{\max}B_{m(\alpha )}}\right)$$

**Figure 4.**
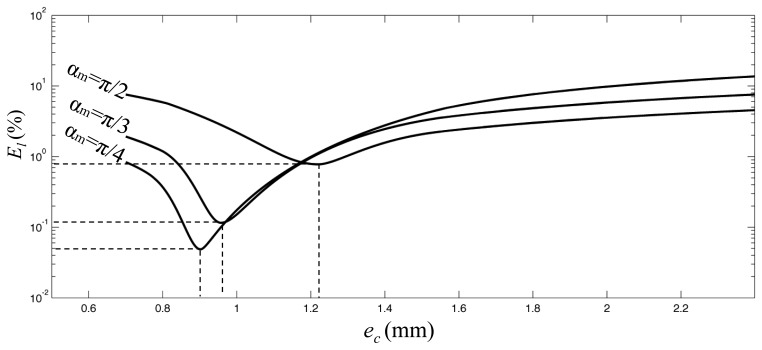
Estimated linearity error *E_l_* versus eccentricity computed for three different range of measure. Plot refers to cylindrical magnet with *M⃗* = [0,1*T*,0], radius *R_c_* = 6 mm, thickness *h* = 6 mm, measurement radius is *r_m_* = 10 mm.

For the sack of clarity, a graphical representation of the linearity error in function of the eccentricity value for three different values of measurement range is presented in Figure 4.

The behavior of the *E_l_* function suggests the possibility of finding optimum values for the parameters which define the geometry of the transducer that minimize the linearity error over a specified range. In particular, for a given magnet geometry it is possible to calculate the combination of measuring radius and eccentricity that provides the optimum linearity over an assigned measuring range. We define the optimum (minimum) value for the linearity error, for an assigned measuring radius with respect to the eccentricity, as:

(7) $$E_{l0}=\underset{e_{c}=[0;r_{m}]}{\min}\left(E_{l}\;(e_{c})\right)$$

and *e_co_* is the corresponding value of the eccentricity.

**Figure 5.**
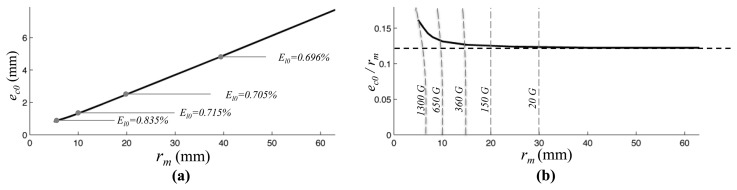
(**a**) Estimated optimum eccentricity in function of the radius of measurement in a range *α_m_* = *π*/2; (**b**) Estimated ratio between the optimum eccentricity and the radius of measurement. Both plots refer to cylindrical magnet with *M⃗* = [0,1*T*,0], radius *R_c_* = 6 mm, thickness *h* = 6 mm, measurement radius is *r_m_* = 10 mm, in a range *α_m_* = *π*/2.

The relation between the optimal eccentricity (*e_co_*) against *r_m_* is represented in Figure 5(a) and in Figure 5(b) the ratio between the optimal eccentricity and the measuring radius is plotted. As it can be observed such ratio tends to a limit constant value that is approximately equal to 0.12 that corresponds to the optimal ratio computed for a magnetic dipole. Figure 5 refers to an example of a cylindrical magnet, however an analogous behavior is observed for annular magnet shape.

### 3.2. Optimization Procedure

**Figure 6.**
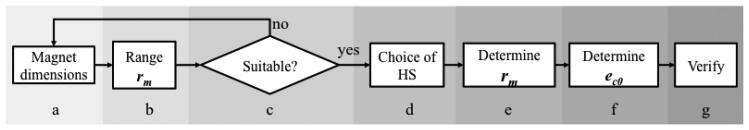
Graphical scheme of the optimization procedure.

On the base of the observations made in the previous section, it is possible to define a procedure that aims at optimizing, over a specific measurement range, the linearity of a sensor that makes use of an assigned magnet. This is particularly interesting to be analyzed since it allows to the designer to choose a magnet of any dimensions according to geometrical requirements of the particular application. Such optimization takes into account the following aspects: (1) linearity of the sensor in a specified angular range and (2) best matching between the measuring range of the employed HS sensor and the actual range of the produced magnetic induction. This last aspect imposes restrictions on the choice of the radius of measurement since the range of measurement of common linear HS is limited to 100– 1200 G. The procedure that is followed for the optimization of the linearity is shown in Figure 6. Assuming that the type of magnet and the measuring range angle *α_m_* are assigned, the procedure go through the following steps: (a) setting initial magnet shape and dimensions on the base of the requirements in terms of encumbrances; (b) establishing, through a plot like Figure 5(b), the range of usable values for *r_m_* that provides a suitable amplitude for *B_m_* (roughly in the range of 100–1,200 G); (c) decide if the range of measurement radius is suitable for the application—if not a different magnet shape has to be considered; (d) choosing a linear HS that roughly provides the measuring radius that is desired for the application, among the commercial available; (e) accurately determine the radius of measurement that provide the best-fit of the range of measurement of the chosen HS; (f) determine *e_co_*with Equation (7); (g) verify the estimated output, linearity error and maximum in the specified range *B_m_*.

### 3.3. Layouts

**Figure 7.**
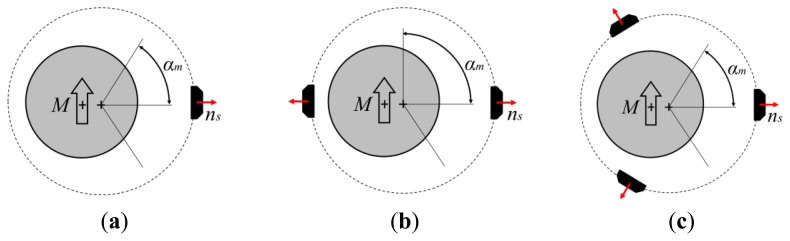
Alternative layouts (**a**) *Layout A* with a single HS allows ranges up to 180°; (**b**) *Layout B* allows 360° of measurement with 2 HS; (**c**) *Layout C* allows 360° of measurement with three HS with improved linearity.

Different layouts can be analyzed and optimized through the procedure defined in the previous section. If the requirements for the sensor allow a limited angular range (0 < *α_m_*< *π*/2), it is possible to employ the *Layout A* represented in Figure 7(a) that includes a single HS sensor. If a continuous measurement over the full turn is required, it is possible to employ alternative solutions represented in Figure 7(b,c). The linearity can be optimized for different ranges *α_m_* according to the type of layout and application requirements. The obtained theoretical performances in term of accuracy (due to linearity error) for a cylindrical magnet are presented in Table 1.

## 4. Experimental Validation

In this section, we present a set of experimental tests that have been conducted for: (1) the validation of the mathematical models presented in Section 2; (2) the verification of the optimization procedure presented in Section 3. A preliminary validation of the proposed model has been conducted through a 3D FEM simulation of the magnet. In such FEM model, the magnet permeability is imposed equal to one. The two models are practically in perfect agreement at convergence.

### 4.1. Methods

**Figure 8.**
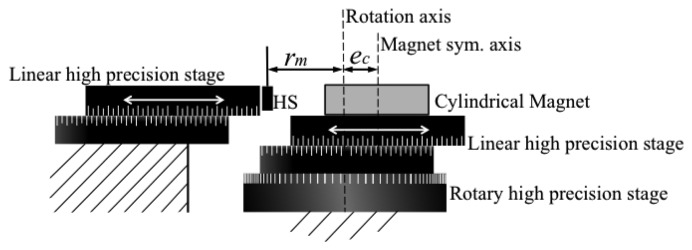
Scheme of the experimental setup employed for the validation of the model and the optimization procedure.

The experimental setup has been arranged by assembling high precision linear and rotary graduated stages. The stages were arranged as represented in Figure 8. A rotary graduated stage and a linear stage connected in series were respectively employed to impose controlled rotations and eccentricity to the magnet. A second linear stage was used to regulate the measuring radius. Tests were conducted using a NdFeB cylindrical magnet (N35) with a residual induction *B_r_* = 1.19 T and an integrated HS commercialized by Honeywell Corp. (model SS495A1 see Table 2 for reference data). Magnet dimensions were chosen among the available commercial standard dimension as *R_c_* = 6 mm and *h* = 6 mm.

High precision, low noise power supply of 5.5 V was provided to power the sensor and a high precision voltmeter (Tektronix DM512) was employed to measure the output signal.

In order to validate the model, we firstly consider the best experimental conditioned measure, in which the eccentricity is set to zero. This is the best experimental condition since the measure is not affected by possible angular error between the magnetization direction and the direction of the imposed eccentricity. In this configuration, the theoretical output the sensor should be a sinusoid according to Equation (4). This test allowed us also to accurately evaluate the orientation and the magnitude of the magnetization vector that is provided by the manufacturer with a large tolerance. Measures have been collected on a full turn by imposing a constant increment to the rotation of the magnet for a total of 33 samples. Each measure was repeated five times with the average being taken, showing a repeatability of 0.18%.

A second test has been conducted with the aim of verifying the performance of a sensor whose geometrical dimensions are set through the optimization procedure presented in Section 3.2. In this case, the acquisition has been done within the established measuring range imposing constant incremental rotations for a total of 33 samples.

### 4.2. Results

**Figure 9.**
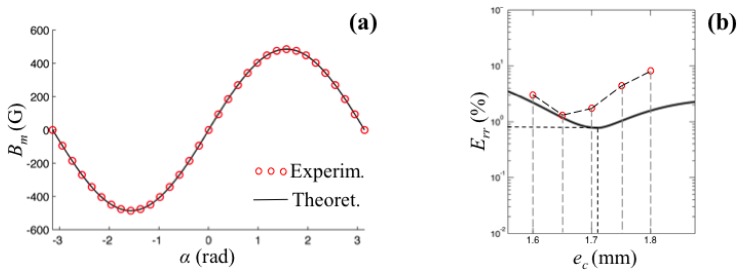
Plot of the experimental results (*r_m_* = 14 mm) (**a**) comparison between theoretical and experimental values of the measured induction field (**b**) comparison between theoretical and experimental optimal values.

The results of the first test are shown in Figure 9(a), where experimental and theoretical data are compared. The maximum error between the theoretical and experimental curves has been evaluated to be the 0.8% of the full scale signal. It is important to underline that the theoretical data were computed on the base of actual residual magnetization that has been reverse-computed through a sinusoidal best fitting of the same experimental dataset. Otherwise, difference of approximately 9% would have been recorded between theoretical and experimental residual magnetization, that is in agreement with the magnet manufacturing tolerances. In order to evaluate the proposed optimization procedure, we set an eccentricity to the rotation of the magnet according to the layout described in Section 3. For the given magnet dimensions, it is estimated (see Table 2) that, in a range of ±*π*/2 and with a measuring radius of 14 mm, the optimum value for the eccentricity is at *e_co_*.

Experimentally, we collect five sets of data for different values of eccentricity chosen in an interval that is roughly centered about the theoretical optimal eccentricity. As it can be observed in Figure 9(b) a difference between the theoretical and experimental optimal values is recorded. Moreover, also considering the actual value of the optimal eccentricity, the linearity of the sensor is about 1.4% against the theoretical value of 0.80%.

This difference is, most probably, not attributable to geometrical fabrication error, but rather it is caused by approximations that have been used in the mathematical model. In order to shade some light on this we have simulated the optimal layout resulting from the numerical optimization with FEM models that take into account the actual relative permeability. Only minor differences, mainly in the magnitude of magnetic induction, between the FEM numerical solutions are noticeable and such a difference does not affect the prediction of linearity. Thus the main source of divergences between theoretical and experimental data can be attributable to non-uniform magnetization of the permanent magnet and to HS accuracy error.

## 5. Applications

**Figure 10.**
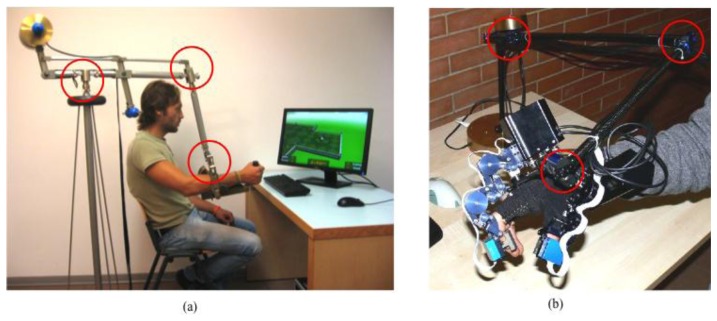
(**a**) Track-Hold: mechanical tracker employed for VR rehabilitation application and (**b**) HFF-Tracker: mechanical tracker employed for tracking portable haptic interfaces.

The new sensing principle and the associated design procedure have been applied for the sensorization of two custom designed mechatronic devices: two different types of mechanical body-trackers, *i.e.*, systems that measure in real-time position and orientation of a part of the body of a user. The two devices were conceived for different applications, the first one is employed for the tracking of the position of the forearm in virtual reality rehabilitation applications ([22]). The second one is a desktop device [21] employed in combination with a wearable hand exoskeleton (described in [26,27]) to measure the position of the user hand. PERCRO laboratory has developed two prototypes of such devices with the aim of commercialization (see Figure 10).

**Figure 11.**
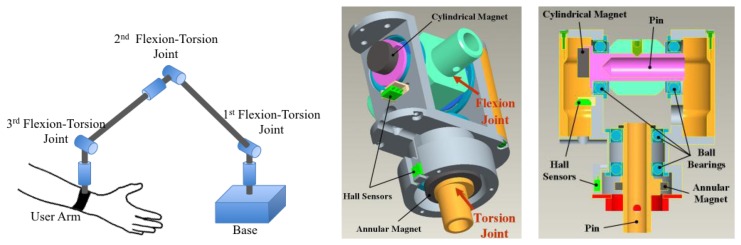
CAD models of the implementation of the Flexion-Torsion joint equipped with two different sensor developed with annular and cylindrical magnet. An equivalent solution has been employed for the Track-Hold.

The sensing requirements for both the devices include characteristics that are well suitable with the sensing principle that have been presented in the previous section such as: high resolution (below tenth of millimeters), low accuracy (in the range of tent of millimeters), limited range of angle measurement (in the range of ±*π*/2), simple cabling (number of cables must be minimized), absolute sensing and low cost. The two developed trackers are based on the same serial kinematics architecture composed by six rotational joints connected by rigid links, as shown in Figure 11. The rotational joints were coupled two by two to form a two degrees of freedom (DoF) unit repeated three times that is named Flexio-Torsion Unit. Each Flexion-Torsion Unit employs two different magnets whose dimensions were chosen according to the available spaces and the need of arranging the wiring that need to pass through the rotation joints.

The sensor dimensions have been selected according to the procedure defined in Section 3, and, before the manufacturing of the hardware prototypes, a characterization using the setup described in Section 4 have been conducted introducing small adjustment to the geometrical parameters. In Table 3 the final dimensions and the performances of and sensors employed for the two types of Torsion-Flexion joints are shown.

## 6. Conclusions

Several application fields of mechatronic technology impose severe requirements on sensors. It is often desirable that linear position and/or shaft angle sensors be compact, low-cost, reliable, highly integrated with the mechanical structure, lightweight and to have minimal complexity. In such cases the employment of off-the-shelf sensors is not possible or economically unviable; thus the sensors are built and designed around the existing mechanical components.

In this paper, we present a novel layout for shaft-angle sensing that makes use of a minimal set of low-cost components that can be easily integrated on existing rotating shafts. Despite the low costs and simple design, the sensor provides a linear output curve over an angular measuring range of up to *π* [in rad] using just one Hall effect sensor (HS). The working principle is based on a diametrically magnetized cylindrical or annular magnet that rotates around an eccentric axis and a HS. One of the main strengths of the novel sensing approach is the possibility of being adapted to assigned encumbrances and spaces without any limitation imposed by standards since the dimensions of the sensor components can be chosen according to design requirements. Moreover, with respect to traditional solutions which employ multiple HS, it features minimal use of sensors and reduction of electrical wiring. This last feature could be particularly relevant for mechatronics and robotic devices that employ serial kinematics. On the other hand the proposed sensing principle is less suitable for high-speed applications since the introduced eccentricity may cause unwanted rotor vibration.

This paper presents a mathematical model for the estimation of the sensor output that is validated against results obtained from laboratory experiments. On the base of the developed model, the layout of the sensing system is optimized for maximizing the linearity over an assigned angular range. In particular, we show how it is possible, for assigned dimension of the magnet, to find a combination of the geometrical parameters that optimizes the linearity of the sensor obtaining an accuracy of approximately 1–2% in the range of up to *π* [rad]. This property is particularly interesting for custom integrated design since it allows one to freely choose the geometrical dimensions of the sensor according to the requirements that are imposed by the particular application.

The new sensing principle has been integrated in the design of two six degrees of freedom mechanical trackers that are employed for: (1) real-time measurement of the hand position for interaction with virtual environments using portable haptic interfaces; and (2) real-time measurement of the forearm position in virtual reality rehabilitation applications.

## Figures and Tables

**Table 1.** Comparison between different optimized layout for an assigned magnet with cylindrical shape (*R_c_* = 6mm, *h_c_* = 6 mm).

**Layout Type**	**Range**	**Range of Optimiz.*α_m_***	**No. of HS**	***e_co_* (mm)**	**Accuracy (%)**
A	[−π/4; π/4]	[−π/4; π/4]	1	0.9	0.05
A	[−π/3; π/3]	[−π/3; π/3]	1	0.95	0.12
A	[−π/2; π/2]	[−π/2; π/2]	1	1.22	0.81
B	No limit	[−π/2; π/2]	2	1.22	0.81
C	No limit	[−π/3; π/3]	3	0.95	0.12

**Table 2.** Table of performances of the sensor Honeywell SS495A1 employed for the experimental validation.

	
**Property**	**Values**	**Unit**	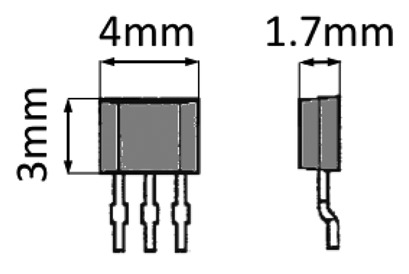

Range of measurement (min)	±600	G	
Output range	0.2–4.8	V	
Power voltage	4.5–10.5	V	
Accuracy	<1%	-	
	

**Table 3.** Table of the employed sensor for the two types of Torsion-Flexion joint developed for the implementation of the two trackers.

**Flexion-Torsion Joint of Track-Hold** (Figure 10-a)					

	**Magnet**	**Mag. Dim.**	**HS**	**Geometry**	**Accuracy**
Torsion Flexion	NdFeB Cylindrical *Br* = 1.10T	*R_a1_* = 7.50 mm *R_a2_* = 10.50 mm *h_a_* = 3.00 mm	SS495A1 Honeywell	*r_m_* = 14.60 mm *e_c_* = 1.67 mm	1.7%
**Flexion-Torsion Joint of Hand Exos Tracker** (Figure 10-b)					

**DoF**	**Magnet**	**Mag. Dim.**	**HS**	**Geometry**	**Accuracy**

Torsion	NdFeB Annluar *Br* = 1.21T	*R_a1_* = 4.70 mm *R_a2_* = 6.70 mm *h_a_* = 2.00 mm	A1321 Allegro	*r_m_* = 10.30 mm *e_c_* = 1.13 mm	1.6%

Flexion	NdFeB Annluar *Br* = 1.21T	*R_c_* = 3.75 mm *h_c_* = 3.00 mm	A1321 Allegro	*r_m_* = 8.50 mm *e_c_* = 1.12 mm	1.5%
